# Different Transcript Patterns in Response to Specialist and Generalist Herbivores in the Wild Arabidopsis Relative *Boechera divaricarpa*

**DOI:** 10.1371/journal.pone.0001081

**Published:** 2007-10-24

**Authors:** Heiko Vogel, Juergen Kroymann, Thomas Mitchell-Olds

**Affiliations:** 1 Department of Genetics and Evolution, Max Planck Institute for Chemical Ecology, Jena, Germany; 2 Department of Entomology, Max Planck Institute for Chemical Ecology, Jena, Germany; 3 Department of Biology, Duke University, Durham, North Carolina, United States of America; Umeå Plant Science Centre, Sweden

## Abstract

**Background:**

Plants defend themselves against herbivorous insects, utilizing both constitutive and inducible defenses. Induced defenses are controlled by several phytohormone-mediated signaling pathways. Here, we analyze transcriptional changes in the North American Arabidopsis relative *Boechera divaricarpa* in response to larval herbivory by the crucifer specialist lepidopteran *Plutella xylostella* (diamondback moth) and by the generalist lepidopteran *Trichoplusia ni* (cabbage semilooper), and compare them to wounding and exogenous phytohormone application.

**Methodology/Principal Findings:**

We use a custom macroarray constructed from *B. divaricarpa* herbivory-regulated cDNAs identified by suppression subtractive hybridization and from known stress-responsive *A. thaliana* genes for transcript profiling after insect herbivory, wounding and in response to jasmonate, salicylate and ethylene. In addition, we introduce path analysis as a novel approach to analyze transcript profiles. Path analyses reveal that transcriptional responses to the crucifer specialist *P. xylostella* are primarily determined by direct effects of the ethylene and salicylate pathways, whereas responses to the generalist *T. ni* are influenced by the ethylene and jasmonate pathways. Wound-induced transcriptional changes are influenced by all three pathways, with jasmonate having the strongest effect.

**Conclusions/Significance:**

Our results show that insect herbivory is distinct from simple mechanical plant damage, and that different lepidopteran herbivores elicit different transcriptional responses.

## Introduction

Plants are challenged by a multitude of herbivorous insects. Most herbivorous insects have a narrow host-range, *i.e.* they feed on plants from one taxonomic family or even a single host species. Therefore, they are often referred to as specialists. Only a minority of insects, so-called generalists, are capable of adapting to the disparate defensive mechanisms of many different plant species, utilizing them as their hosts. To ward off these pests, plants have evolved an arsenal of defensive traits. These traits include preformed physical or chemical barriers, as well as inducible defenses [1]. Inducible defenses are activated upon herbivore attack, and involve three conceptual phases, pest recognition, signal transduction, and deployment of defenses. Plants may recognize herbivorous insects by the mechanical damage herbivores exert on their host plants, and/or by chemical cues originating from the insects' surface or digestive fluids [2]–[4]. Open wounds lead to water loss and serve as a potential entry point for pathogens. Therefore, herbivore-associated wounding may elicit pathways that also are induced by pathogens or drought [5], [6].

Several phytohormone-mediated signal transduction pathways control induced defense responses, and include the jasmonic acid (JA), ethylene, and salicylic acid (SA) pathways. Their relative contribution depends both on the host plant under study, and on the type of biotic interaction. Cellular responses to phytohormone signals are highly regulated and complex, and both positive and negative cross-talk occurs between different phytohormone-mediated signal transduction pathways [7]–[14]. JA, for example, is widely accepted to be a key factor in the regulation of wound and drought responses, and there is extensive information on the role of the jasmonate pathway in resistance to insects [15]–[22]. However, both JA-dependent and JA-independent wound signaling pathways have been described in *A. thaliana* and tomato [23], [24]. Furthermore, some inducible plant defenses depend on the concerted action of JA and SA or ethylene, and positive and negative interactions have been described both at the physiological and molecular level [25]–[33].

Expression profiling provides a useful description of transcriptional responses of many genes to various experimental conditions, including challenges imposed by insect herbivores [6], [22], [34], [35]. Several studies have investigated whether different insect herbivores elicit species-specific transcriptional responses. However, analyses of different plant models led to different conclusions. In the case of *Nicotiana attenuata*, different transcript response patterns have been reported for different lepidopterans [34], while for *Arabidopsis thaliana* nearly identical responses to lepidopteran herbivory were found when challenged with *Pieris rapae*, a crucifer specialist, or Egyptian cotton worm (*Spodoptera littoralis*), a crucifer generalist [22].

We investigated *Boechera divaricarpa* transcript patterns in response to two lepidopteran herbivores, the specialist diamondback moth (*Plutella xylostella*), and the generalist cabbage semilooper (*Trichoplusia ni*). *B. divaricarpa* is a close relative of *A. thaliana*, sharing a common ancestor with *A. thaliana* about 10 million years ago [36]. *B. divaricarpa* is a perennial species, and, thus, experiences multiple selective episodes by a diverse herbivore community during its life cycle. *Trichoplusia ni* (*T. ni*) is the third most damaging pest of cultivated Cruciferae [37], and is established worldwide, except for Australia and the tropics. Its broad host range includes tomato, lettuce, potato, beans, maize, cotton, and other plants. This pest is difficult to control in agricultural settings due to population explosions and larval resistance to several insecticides [38], [39]. *Plutella xylostella* (*P. xylostella*), a crucifer specialist, is one of the most widespread lepidopteran species, and often seriously damages cruciferous crop plants, especially in the tropics [40]. In many countries, this pest has developed resistance to a broad array of insecticides, including the *Bt* toxin [41]–[43]. *P. xylostella* has a very patchy feeding mode, exerting damage on several smaller areas of plant leaves. In contrast, *T. ni* mostly feeds from the leaf edges, leading to larger and more localized damage patterns.

In this study, we investigate transcript patterns in the context of insect herbivory, and introduce path analysis as a novel approach to determine similarities and disparities in gene expression patterns between *T. ni*- and *P. xylostella*-induced responses, wounding, and the exogenous application of the phytohormones JA, SA, and ethylene.

## Materials and Methods

### Plant Materials and Growth Conditions

*A. thaliana* seeds (ecotype Columbia) were obtained from the Nottingham *Arabidopsis* Stock Center. *Boechera divaricarpa* VP#9 seeds were collected in Vipond Park, Montana (Coordinates: 45° 40′ 57″ N, 112° 53′ 53″ W). Seeds from both plant species were sown on a Mini-Tray: vermiculite (3:1) soil mix (Einheitserdenwerk, Fröndenberg, Germany) and cold stratified for 7 days at 4°C. Afterwards, plants were moved to ventilated growth rooms with constant air flow and 40% humidity at 23°C. Plants were grown at a distance of 30 cm from fluorescent light banks with four bulbs of cool white and four bulbs of wide spectrum lights at a 14 h light/10 h dark photoperiod. Seeds germinated in 2–3 (*A. thaliana*) and 6–7 (*B. divaricarpa*) days. Grow domes were removed after 5 days under lights and plants were fertilized once with 1 ml of Scotts Peters Professional Peat Lite Special 20N∶10P∶20K with trace elements and 1 liter water per flat, added to the bottom of the tray. Approximately 6 days after germination, plants were transferred to individual pots (7.5×7.5 cm^2^) and were grown for 22 days (*A. thaliana*) or 40 days (*B. divaricarpa*) under strict light, temperature and humidity control.

### Insect Growth Conditions

Diamondback moth (*Plutella xylostella*) eggs (G-88 strain) were originally obtained from the New York State Agricultural Experimental Station (Geneva, NY), and a colony was maintained in Jena. Cabbage semilooper (*Trichoplusia ni*) eggs were obtained from Benzon Research (Carlisle, PA). Larvae from both species were reared on a wheat germ based artificial diet according to published procedures [44] at 27°C and 16 h light/8 h dark cycles. Herbivory screens were performed with fourth-instar *P. xylostella* and third-instar *T. ni* larvae.

### Plant Treatments

All induction experiments were performed 4 (*A. thaliana*) or 7 weeks (*B. divaricarpa*) post germination. All plants were at a vegetative growth stage and pre-bolting. For each experiment, control plants were included and subjected to the same environmental conditions (except for the respective experimental trigger) as treated plants. **Wounding** was performed with forceps and scissors. Rosette leaves were damaged several times every 30 minutes during experiments, inflicting damage on a leaf area comparable to insect herbivory at the various time points. Insect **herbivory screens** were carried out with two larvae per rosette leaf. Exogenous phytohormone treatment followed published procedures to ensure compatibility with previous research [45], [46]. For **MeJA treatments**, 400 µl of 3% (v/v) MeJa (Sigma-Aldrich, Seelze, Germany), dissolved in ethanol, were mixed with Lanolin (Fluka, Buchs, Switzerland) and applied to transparent grow domes. Grow domes were placed onto trays and sealed. Control plants were treated in the same manner, except that 1% (v/v) ethanol without MeJA was used. For **SA treatments**, plants were sprayed evenly with a 1 mM solution of salicylic acid (Sigma-Aldrich), and each plant received approximately 300 µl spray. Afterwards, grow domes were sealed to the trays. For **ethylene treatments**, 400 µl of a 10mg/ml solution of Ethephone (2-chloroethyl-phosphonic acid; Riedel-de Haën, Seelze, Germany) dissolved in ethanol was mixed with Lanolin and applied as described above. Untreated controls were subjected to the same environmental conditions but without addition of inducers. For each treatment, leaf tissue was harvested after 0.75, 3, 9 and 24 h; in addition, control leaves were also harvested at the start of each experiment (0 h). Leaf material was immediately frozen in liquid nitrogen and stored at −80°C. The numbers of biological replicates ranged from 2–4, each with 1–3 technical replicates.

### Suppression Subtractive Hybridization (SSH)

*B. divaricarpa* cDNAs corresponding to genes differentially regulated by *P. xylostella* herbivory were obtained with the PCR-Select Subtractive Hybridization kit (BD Clontech, Heidelberg, Germany) according to the manufacturer's instructions. Experimental and control samples were processed simultaneously to reduce false positives. Both forward and reverse subtractive hybridizations were performed. To identify genes with increased transcript levels after herbivory, poly(A)^+^ mRNA from wounded plants was used as a ‘driver’ and poly(A)^+^ mRNA from insect-treated plants as a ‘tester’. Likewise, ‘driver’ and ‘tester’ were reversed to obtain genes with reduced transcript levels after herbivory. In both cases, subtracted cDNAs were subjected to two PCR cycles to allow for normalization of cDNA populations. PCR products from both primary and secondary PCR reactions were analyzed according to the manufacturer's instructions. Subtracted cDNAs were cloned into the pCRII TOPO T/A vector (Invitrogen, Karlsruhe, Germany) according to the manufacturer's instructions. Transformations were performed with *E. coli* ELECTROMAX DH5α-E cells (Invitrogen).

### Sequencing and Sequence Analyses

Plasmid minipreparation was performed according to standard procedures. In total, 1,500 clones were generated and sequenced from both ends on an automated Applied Biosystems 3700 DNA Analyzer using BigDye terminators version 2.0 (PE Applied Biosystems Inc., Weiterstadt, Germany). Sequences were analyzed with the DNAstar software package (DNASTAR Inc., Madison, WI), and edited manually. Genes were identified using BLAST [47]. A series of filtering steps was applied to identify and remove reads that did not contain any or very short inserts. Each sequence was edited to correct sequencing ambiguities, remove primer sequence and exclude chimeric clones. Chimeric clones were identified as those reads with adaptor sequences within reads, and they were also removed. Sequences were assigned to functional classes based on that of its most closely related *A. thaliana* sequence using BLAST, and the gene families available at The Arabidopsis Information Resource (TAIR; www.arabidopsis.org) (Table 1, Table S1).

**Table 1:** GO Annotation of *B. divaricarpa* Genes Identified by Suppression Subtractive Hybridization.

Biological process	No. of genes
Cell growth, division & development	27
Cellular metabolism	105
Energy pathways & electron transport	38
Protein synthesis, folding & modification	59
Transcription & translation	44
Cellular communication & signalling	26
Transport & homeostasis	40
Defence, stress response & detoxification	130
Unknown genes	141
Functional category (TAIR)	No. of genes
Transferase activity	40
Other enzyme activity	110
Hydrolase activity	90
Protein binding	21
DNA or RNA binding	23
Other binding	42
Structural molecule activity	20
Kinase activity	23
Transcription factor activity	31
Receptor binding or activity	10
Nucleotide binding	11
Transporter	23
Other molecular function	25
Molecular function unknown	141
Total no. of unique genes	610

### cDNA Macroarrays

We prepared nylon membrane custom macroarrays with cDNAs and controls from several sources. Approximately 450 *B. divaricarpa* cDNAs were obtained from our subtractive libraries. We also obtained a collection of 110 *A. thaliana* clones from the Arabidopsis Biological Stock Center (ABRC, University of Ohio, Columbus, OH), preselected for differential regulation upon environmental stress. These clones were re-sequenced to confirm their identity, and approximately 20% were discarded due to incorrect clone assignments. For a variety of clones from both sources, PCR was used to obtain homologous sequences from both *B. divaricarpa* and *A. thaliana*. Thus, 58 genes were represented by cDNAs from both species.

Plasmid inserts were amplified by PCR with flanking vector-specific primers. Product size, quality and quantity were verified by agarose gel electrophoresis. PCR products were purified using 96-well Sephadex plates, transferred to two 384-well microtiter plates, and concentrated to ca. 100–200 ng/µl. Microtiter plates included several additional controls; pCRII TOPO vector (Invitrogen), M13 universal forward and reverse primers, oligo(d)-T (Stratagene, La Jolla, CA), poly(A) DNA, Tris-EDTA buffer, human beta-actin, salmon sperm DNA, and PCR counterparts of 10 different artificial RNA spike controls from the Alien cDNA spot control system (all Stratagene). To each well, an equal volume of 1 N NaOH/100 mM EDTA was added, and samples were denatured for 10 min at 37°C. Samples were arrayed in duplicate on nylon membranes (Nytran SuperCharge; Whatman, Dassel, Germany) with a 384 pin tool attached to a Biomek 2000 robotic platform (Beckman-Coulter, Krefeld, Germany), and DNA was cross-linked by UV-irradiation at an integrated intensity of 120 mJ cm^−2^ using a BioRad UV crosslinker (BioRad, München, Germany).

### RNA Isolation and Probe Preparation for Macroarrays

Leaf material was ground to a fine powder in liquid N_2_, and total RNA was isolated using the TRIzol Reagent (Invitrogen) according to the manufacturerś protocol. A second purification step was performed with RNeasy columns (Qiagen, Hilden, Germany). A DNAse treatment was included prior to the second purification step to eliminate any contaminating DNA. DNAse was afterwards inactivated according to the manufacturer's recommendations (Ambion, Austin, TX). RNA integrity was verified on non-denaturing agarose gels. RNA quantity was determined photospectrometrically. Probe labeling was performed with the Strip-EZ kit (Ambion) and α-^33^P-dATP. In brief, approximately 5 µg total RNA were mixed with random decamer and oligo-dT primers, and the Alien RNA Spike mix (Stratagene), and heated to 65°C for 10 min. After cooling at ambient temperature for 5 min, RT buffer, dNTPs, α-^33^P-dATP (2 µl of a 3,000 Ci/mmol solution), 400 U reverse transcriptase (custom blend of Superscript III (Invitrogen) and Omniscript (Qiagen)), and water were added to a final volume of 20 µl. Reverse labeling was performed in a thermal cycler at 25°C for 10 min, 37°C for 1 h and 50°C for 1 h. After purification on sephadex G-25 columns (Amersham), samples were used immediately for hybridization. In total, more than 250 hybridizations were performed, of which 200 were of sufficient quality for further analyses.

### Hybridization and Detection

Membranes were pre-hybridized with 5 ml of Ultra-Hyb hybridization solution (Ambion) at 42°C for 1 h. Samples were denatured at 100°C for 3 min, left at ambient temperature for 5 min, and added to the pre-hybridization solution. Hybridizations were carried out o/n at 42°C. Membranes were washed twice in 2× SSC, 0,5% SDS at 42°C for 20 min and once with 0,5× SSC, 0,5% SDS at 50°C for 30 min. Membranes were wrapped in clear plastic bags and exposed to Phosphoimager screens for 24–48 h. Membranes were stripped with Strip-EZ kit (Ambion) according to the manufacturer's protocol, and re-used up to 5 times. Hybridization signals were recorded with a Storm 840 Phosphoimager (Amersham Biosciences, Freiburg, Germany) at the 50 µm level. Raw data analysis, signal quantification and background correction were performed with Arrayvision version 6.0 (Imaging Research, Ontario, Canada).

### Normalization and Statistical Analysis

To control for variation in reverse transcription efficiency, labeling and hybridization, we utilized the Alien cDNA spot control system (Stratagene), which consists of 10 different artificial RNA spike controls and their respective PCR probe counterparts.

Each array contained 74 alien spike controls with foreign DNA (PCR products) which were hybridized (spiked in the plant RNA samples) with a mixture of their respective mRNAs at final concentrations ranging from 0.002–1.0 ng/µl. Information from these controls was used to develop a predictive equation to normalize expression levels within and among arrays. First, all data were log transformed: *Y_i_* = Log10(*X_i_*+1.0), *X_i_* is the untransformed signal from the i-th spot, *Y_i_* is log transformed hybridization intensity, and 1.0 is added to ensure positive data. Then we developed a predictive equation using spike controls to relate observed hybridization signal to known DNA target concentration: *Z_i_* = *a*+b_1_*Y_i_+b_2_*Y_i_^2^, where *a*, *b_1_*, and *b_2_* are the intercept, linear, and quadratic regression coefficients from a second order least squares regression model, and *Z_i_* is the normalized signal intensity. This procedure was followed for each array, using known concentrations and observed hybridization intensities for spike controls to develop an array-specific predictive equation, which then enabled quantitation of transcript levels of all plant genes on each array. This approach provides an empirically verified linear relationship between signal intensity and transcript level for each array. Finally, trancript levels from each array were multiplied by a constant so that mean transcript level of spike controls equaled 1.0 on each array.

Further statistical analyses were performed with Systat Version 10 (SPSS Inc., Munich, Germany). We conducted nested analysis of variance to identify statistical effects of treatments, biological replicates within treatments, and arrays within biological replicates. To reduce analytical complexity, for each gene on the array we analyzed the arithmetic mean of replicated spots within arrays. For each gene, we used the following statistical model:
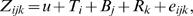
where *u* is the grand mean, *T_i_* is the effect of the i-th treatment, *B_j_* is the effect of the j-th biological replicate within the i-th treatment, and *R_k_* is the effect of the k-th technical replicate array within the j-th biological replicate. Treatments are fixed effects, whereas biological and technical replicates are random effects. To address biological hypotheses we performed statistical tests of treatment differences controlling for biological variability, quantified in ANOVA by the ratio of mean squares, MS_trt_/MS_biol_.

Transcription profiling involves statistical tests on hundreds or thousands of genes, and therefore requires adjusted significance thresholds to control for these multiple tests. We inferred statistical significance in terms of the false discovery rate using Q-values [48]. In brief, a Q-value of 0.05 means that among those features that are called significant, a proportion of 5% will be false positives. Because genes on this custom array were highly enriched for herbivory-related genes, we used a Q-value threshold of 0.10 to infer statistically significant differences among treatments. To infer statistical significance among biological replicates we used the ratio of mean squares, MS_biol_/MS_tech_, with a Q-value threshold of 0.001.

### Path Analysis of Hormone Response Coefficients

**Figure 1:**
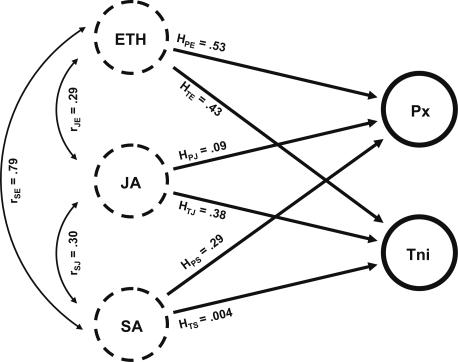
Path Diagram of Insect-Induced Transcriptional Responses. Changes in gene expression which follow wounding or herbivory are causally attributable to the SA, JA, and ethylene regulatory pathways. Three causal variables (ETH, JA, SA; dashed circles) are shown on the left side of the figure, intercorrelated (curved, double headed arrows) due to unknown regulatory and physiological factors (no causal relationship is assumed among these predictor variables). Two response variables (closed circles), DBM (for *Plutella xylostella*), and TNI (for *Trichoplusia ni*), are shown on the right side of the figure; wounding is not shown to reduce the figure's complexity. Causal influences are indicated by straight, single headed arrows, which are standardized partial regression coefficients. The input data for these analyses are ratios quantifying induced versus control expression levels at nine hours for the 212 genes showing statistically significant changes in gene expression.

Path analysis and related methods [49] examine statistical consequences of biological models of causal relationships among variables. Here we assume that the changes in gene expression which follow wounding or herbivory are causally attributable to the SA, JA, and ethylene regulatory pathways. This causal model is formalized in Figure 1: three causal variables (ETH, JA, SA; dashed circles) are shown on the left side of the figure. They are inter-correlated (curved, double headed arrows) due to unknown regulatory and physiological factors, and no assumptions are made regarding possible causal relationships among these predictor variables. (These causal variables may be inter-correlated, but the reason for these correlations is not examined in these path analyses.) Causal influences are indicated by straight, single headed arrows, which are standardized partial regression coefficients. Response variables (*P. xylostella* and *T. ni*, as well as WND, which is not shown in Figure 1 in order to improve clarity) are controlled by ETH, JA, SA, as well as uncorrelated residual effects.

The hormone response coefficient, *H*, is a standardized partial regression coefficient. First, analyses begin with normalized signal intensities (*Z_ijk_*; defined in the “Normalization and Statistical Analysis” section). Next, using the mean signal intensity for each gene (averaged across biological replicates), we compute the ratio of expression in a given treatment at nine hours to expression in the control treatment at nine hours. Finally, for a scale-independent comparison of several variables, changes in gene expression data are expressed in standard deviation units, to control for different levels of variability among traits, and the correlations (Table 2) and path coefficients (Table 3) are computed for each induction treatment. These partial regression coefficients quantify the effect of a single causal variable (such as effects of the SA pathway), controlling for correlations among SA, JA, and ethylene pathways. These analyses only use genes which show significant changes in gene expression in order to reduce unrelated variation attributable to genes which do not show significant responses to these experimental treatments.

**Table 2:** Correlation of Changes in Gene Expression among Three Causal (ETH, ethylene; SA, salicylic acid; JA, jasmonic acid) and Three Response Traits (DBM, *P. xylostella* herbivory; TNI, *T. ni* herbivory; WND, wounding).

**DBM**	1.00					
**TNI**	0.54	1.00				
**WND**	0.50	0.35	1.00			
**ETH**	0.78	0.54	0.45	1.00		
**SA**	0.73	0.46	0.45	0.79	1.00	
**JA**	0.33	0.50	0.42	0.29	0.30	1.00
	**DBM**	**TNI**	**WND**	**ETH**	**SA**	**JA**

All p<0.001 with Bonferroni correction.

Analyses are based on mean changes in expression between induction and control treatments at nine hours for 212 genes (see Materials and Methods for details).

**Table 3:** Correlations (Corr) and Hormone Response Coefficients (HRC) between Causal (ETH, SA, JA) and Response Variables (DBM, TNI, WND).

Effect	DBM			TNI			WND		
	Corr	HRC	P	Corr	HRC	P	Corr	HRC	P
ETH	0.780	0.530	<0.001	0.540	0.433	<0.001	0.450	0.204	<0.05
SA	0.730	0.287	<0.001	0.460	0.004	n.s.	0.450	0.201	<0.05
JA	0.330	0.091	<0.05	0.500	0.378	<0.001	0.420	0.299	<0.001

### Accession Numbers

Sequence data have been deposited in the EMBL/GenBank libraries (accession numbers DQ226545–DQ226990).

## Results

### Identification of Herbivory-related Candidate Genes by Suppression Subtractive Hybridization

We used PCR-based suppression subtractive hybridization (SSH) as one source to obtain herbivory-related candidate genes. *B. divaricarpa* plants were either wounded several times with forceps and scissors or subjected to herbivory by *P. xylostella* larvae. Both forward and reverse subtractions were carried out to identify genes that were differentially regulated between both treatments. More than 1,000 clones were isolated. Sequencing was performed on both ends to enable identification of chimeric clones. Both chimeric and very short clones were excluded from further analysis. Sequences were assembled with DNASTAR using moderately stringent parameters (*i.e.*, match size was chosen to be at least 40 nucleotides, and match percentage was 90). With these parameters we obtained 730 contigs. One fifth of these contigs contained sequences from more than one clone. In some cases, these clones represented clearly distinguishable members of gene families. In other cases, sequences from several clones within one contig were identical or nearly identical (except for occasional single-nucleotide polymorphisms which may have been caused by PCR artifacts), suggesting large differences in transcript abundance between treatments for these genes.

Most abundant were sequences representing fragments from thionin genes. Here, visual inspection identified a gene family whose members were present in different proportions. Also, highly abundant were fragments from genes encoding chlorophyll A–B binding proteins of the LHCB1 and LHCB2 types, and genes encoding lipid-transfer proteins. In these cases, the level of sequence polymorphism also suggested that the clones were derived from different members of gene families. In other cases of highly abundant sequences, *e.g.* for a plant defensin, an elongation factor 1-alpha (EF-1α), a strictosidine synthase, or a vesicle-associated membrane family protein (VAMP), *B. divaricarpa* clones displayed no or very few differences, while the *A. thaliana* genome harbored several related genes. Finally, for a myrosinase-associated protein (At3g14210), a glycine-rich protein (At3g07560), a protease inhibitor of the Kunitz family (At1g72290), a defective embryo and meristems (dem) related protein (At4g33400), a lipase (At2g42690), and two expressed proteins (At1g16080, At4g15790), different *B. divaricarpa* clones were (nearly) identical and matched single genes in *A. thaliana*.

For 610 *B. divaricarpa* cDNAs, *A. thaliana* locus identifiers corresponding to the best BLAST matches were obtained, and classified using the gene ontology (GO) annotation tool from the TAIR webpage (http://www.arabidopsis.org). These annotations were then grouped according to biological processes and functional categories (Table 1). The largest group (23%) belonged to genes of unknown function. Genes encoding proteins important in cellular defense, detoxification and stress response were found to constitute the second most prevalent class of ESTs (21%), including endochitinases, proteinase inhibitors, beta-1,3-glucanases, glutathione S-transferases, peroxidases, superoxide dismutases, and cytochrome P450 mono-oxygenases. The third large group of genes (17%) coded for proteins involved in cellular metabolism, *e.g.* acyl-CoA synthetase (At2g47240, fatty acid biosynthesis), epoxide hydrolase (At4g02340, aromatic compound metabolism), pullulanase (At5g04360, carbohydrate metabolism), and cystathione gamma lyase (At1g64660, amino acid metabolism). A complete list of the TAIR identifiers corresponding to *B. divaricarpa* best BLAST matches and their GO categorization can be found in Table S1.

### Custom Array Generation and Hybridization

We used the genes identified with suppression subtractive hybridization to design an array highly enriched in *B. divaricarpa* cDNAs with insect-responsive expression. We also added genes known from the literature to be differentially expressed in *A. thaliana* under several stress regimes, like drought stress, wounding, insect feeding, pathogen infection and phytohormone treatments [6], [28], [50], [51]. The array consists of 1534 elements representing ∼700 cDNAs spotted in duplicate from *A. thaliana* (Col-0) or *B. divaricarpa*. Of these, 454 cDNAs are derived from *B. divaricarpa* (of which 49 showed no clear match to the *A. thaliana* database), and 263 cDNAs from *A. thaliana* (Col-0). 58 cDNAs were amplified and spotted from both plant species to allow between species comparisons of transcript levels for a limited set of genes. Table S2 contains information about the macroarray setup.

To control for variation in reverse transcription efficiency, labeling and hybridization, we utilized artificial RNA spike controls and their respective PCR probe counterparts. Information from these controls was used to develop a predictive equation to normalize expression levels within and among arrays. We conducted nested analysis of variance to identify statistical effects of treatments, biological replicates within treatments, and arrays within biological replicates. Data for all macroarray hybridizations can be found in Table S3. In general, we aimed at obtaining data from at least 3 biological replicates. However, in some cases hybridizations yielded poor results, and the respective data were excluded from further analyses.

We first compared differences in transcript abundance between 20 treatments, all performed with *B. divaricarpa* (Table S4). These treatments include controls at 0.75, 3, 9, and 24 h, wounding at 0.75, 3, and 9 h, *P. xylostella* herbivory for 0.75, 3, 9, and 24 h, *T. ni* herbivory for 9 and 24 h, JA for 3, 9, and 24 h, SA for 9 and 24 h, and ethylene for 9 and 24 h. 212 cDNAs showed significant changes in expression among these times and experimental treatments. Because this custom array is focused on genes that were known *a priori* to be induced by herbivory, we used a Q-value threshold of 0.10 to infer statistically significant differences among treatments. Therefore, about 21 cDNAs (10%) are expected to represent false positives, and 191 cDNAs are expected to show genuine changes in gene expression. Furthermore, Table S4 is sorted so that false positives are likely to appear at the bottom of the list, and highly significant genes are listed first.

Because of partial redundancy, these 212 differentially transcribed cDNAs correspond to approximately 190 genes. Redundancy is caused by clones obtained by cDNA subtraction and representing different portions of the same or highly similar genes, and also by duplicates spotted from both *A. thaliana* and *B. divaricarpa*. Typically, transcript patterns of these redundant genes are highly correlated. A notable exception involved four thionin cDNAs obtained from *B. divaricarpa* by cDNA subtraction. These clones represent paralogs of *A. thaliana* thionins. For three *B. divaricarpa* thionin cDNAs, transcript patterns were highly correlated with one another (r>0.94), while the fourth showed a distinct expression profile not correlated with any of the other thionin cDNAs.

**Figure 2:**
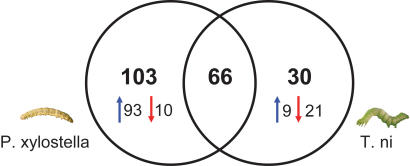
Venn Diagram of the numbers of overlapping and non-overlapping *Boechera* genes up-regulated (blue arrows) and down-regulated (red arrows) in response to herbivory by *T. ni* and *P. xylostella* caterpillars.

Within this set of cDNAs with statistically significant differential expression, we categorized genes as differentially regulated between *P. xylostella* and *T. ni* based on an arbitrary fold-cutoff of 1.3 for “upregulated” and 0.7 for “downregulated” genes in comparison to wounding. 9 h after induction, 160 cDNAs showed higher and 8 cDNAs lower transcript abundance in *P. xylostella*-treated plants compared to wounded plants (Figure 2). In contrast, herbivory by *T. ni* led to higher transcript abundance for 69 cDNAs, but 27 cDNAs had lower transcript abundance (Table S4). Hence, these data suggest that *P. xylostella* and *T. ni* trigger different transcriptional responses in the investigated plants.

### Path Analysis

For a direct comparison of the transcriptional responses exerted by the two different insects in this study, and to examine causal influences of the phytohormones JA, ethylene and SA, we conducted a path analysis. Path analysis is a statistical method closely related to multiple regression, and designed to examine statistical consequences of biological models of causal relationships among variables [52], [53]. Data for analysis of hormone response coefficients is based on significant expression changes for 212 cDNAs in relationship to unmanipulated controls, with the genes as the unit of replication. Highly significant correlated changes in gene expression are observed among three causal and three response traits (Table 2; all p<0.001 with Bonferroni correction for multiple tests). Correlations among hormone responses were uniformly positive. Responses to ethylene and SA showed the strongest positive correlations (r = 0.79). Although SA and JA pathways are often considered to act antagonistically [8], [9], [54], nevertheless, their overall correlated expression changes were significantly positive.

**Figure 3:**
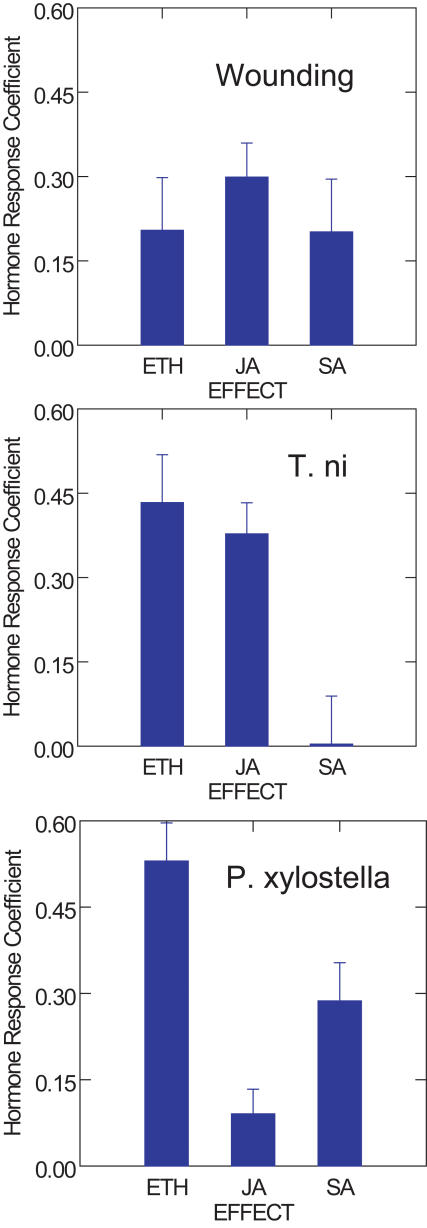
Hormone Response Coefficients for Wound-, *T. ni*-, and *P. xylostella*-Induced Changes. Hormonal control differs significantly between *T. ni* and *P. xylostella* (P<0.01 by ANCOVA). Wound-induced changes in transcript levels are influenced by all three hormonal pathways, although JA has the strongest direct effects.

The correlations between hormone responses and insects or wounding are attributable to a combination of direct and indirect causal pathways, and hormone response coefficients allow us to identify causal influences within these composite correlations. For example, the positive correlation between SA induction and *T. ni* responses (0.46, Table 2) can be partitioned into the summation of direct and indirect pathways:
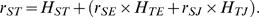



Direct effects of the SA pathway on *T. ni* - induced changes (*H_TS_* = 0.004), are negligible, whereas the total indirect effects (*r_SE_*×*H_TE_+r_SJ_*×*H_TJ_* = 0.456) result primarily from the strong correlation between SA and ethylene responses, which in turn have direct effects on induced responses to *T. ni*. Thus, Figure 1 and Table 3 show that *H_PS_* is 0.287 for *P. xylostella*, but *H_TS_* = 0.004 for *T. ni*. This means that transcriptional responses to *P. xylostella* are equivalent to 28.7% of SA-induced effects, but in *T. ni* these transcriptional changes correspond to only 0.4% of salicylate-induced changes. Thus, transcriptional responses to *P. xylostell*a are primarily determined by direct effects of the ethylene and SA pathways, while *T. ni* induced changes are attributable primarily to direct effects of the ethylene and JA pathways. Hormonal control of herbivore-induced changes differs significantly between *P. xylostella* and *T. ni* (P<0.01 by analysis of covariance; Table 4). Finally, wound-induced changes in gene expression are influenced by all three pathways, although JA has the strongest direct effects (Figure 3).

**Table 4:** Analysis of Covariance for Hormonal Control of Insect-Induced Changes.

Source	df	Mean Squares	F-ratio	P
Insect	1	0.452	4.95	0.0266
ETH	1	7.995	87.53	0.0000
SA	1	0.961	10.53	0.0013
JA	1	3.452	37.79	0.0000
Insect * ETH	1	0.598	6.55	0.0108
Insect * SA	1	0.927	10.15	0.0016
Insect * JA	1	0.823	9.01	0.0028
Error	416	0.091		

### Comparison of Transcript Patterns between *A. thaliana* and *B. divaricarpa*

Do transcriptional responses differ between plant species? For 58 genes, hybridization targets from both plant species, *B. divaricarpa* and *A. thaliana*, were spotted onto the array, thus permitting us to include a comparison of homologous *vs.* heterologous hybridization signal strengths for both species in our statistical models. We used these data to estimate the number of genes that showed a) differences in mean transcript level between species, and b) differences in transcript patterns between species. Comparisons were restricted to seven treatments with sufficient replication, *i.e.*, with at least three independent biological replicates each. These treatments involved controls at 9 and 24 hours, insect herbivory at 9 and 24 hours, JA treatment at 24 hours, SA treatment at 9 hours, and wounding at 9 hours. We used mixed-model ANOVA and tested for species effect, treatment, interaction between treatment and species effects, as well as for biological replicates within interaction of treatments and species:
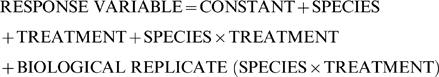



F ratios were calculated as MeanSquare_(SPECIES×TRT)_/MeanSquare_BIOL_REP(SPECIES×TRT)_. Statistical significance in ANOVA was inferred by Q-statistics to minimize false positives; a Q-value of 0.05 was used as a cut-off.

Surprisingly, we detected statistically significant differences in mean transcript levels for only 10 pairs of targets (corresponding to *A. thaliana* genes At1g11840, At1g64720, At1g72290, At1g76180, At2g02990, At2g24420, At4g33150, At4g39090, At5g24770, and At59310; see: Table S5). Only At1g64720, encoding a presumptive membrane protein of unknown biological function, displayed a higher mean transcript level in *A. thaliana*. For the other nine genes, mean transcript levels were between 36% (At4g39090) and 141% (At5g59310) higher in *B. divaricarpa* than in *A. thaliana*. The respective *B. divaricarpa* sequences were re-examined by BLAST against the *A. thaliana* database (http://www.arabidopsis.org), and found to encode proteins of diverse function. In most cases, only one or two strong (and sometimes, additional clearly weaker) BLAST hits were detected, indicating that most of the respective hybridization signals were attributable to single genes when hybridizing with *A. thaliana* cDNA. Two notable exceptions were At5g24770 and At5g59310. At5g24770 encodes a vegetative storage protein [55], [56] and is present in two nearly identical copies. At5g59310 encodes a lipid transfer-like protein, and is a member of a small gene family in *A. thaliana* [57], [58]. In contrast, several of these genes (corresponding to At1g11840, At1g72290, At2g02990, At2g24420, At5g24770, and At5g59310) were present in more than one copy in our *B. divaricarpa* subtractive library, sometimes with substantial sequence polymorphism between copies. This suggests that the respective genes are present in several allelic copies or may be members of multigene families in *B. divaricarpa*. Therefore, higher mean transcript levels in *B. divaricarpa* may be at least partially attributable to the contribution of several gene family members.

Statistically significant differences in expression patterns were detected for only two target pairs, corresponding to At3g62600 and At4g31500 (Table S5). At3g62600 encodes a DNAJ heat shock protein. At4g31500 encodes CYP83B1, a cytochrome P450 enzyme involved in the biosynthesis of aromatic and indole glucosinolates. In total, 12 out of 58 genes showed statistically significant differences between species. This corresponds to a proportion of ca. 21%, and suggests that transcriptional responses to the investigated triggers are largely conserved between *A. thaliana* and *B. divaricarpa*.

## Discussion

### Differential Gene Expression in Response to Insect Herbivory

We used suppression subtractive hybridization and transcript profiling to identify genes related to insect herbivory. We conducted transcript profiling with custom-generated macroarrays, and investigated both *A. thaliana* and its wild relative *B. divaricarpa*. In general, the results obtained with different approaches support each other. Among those genes that were identified as herbivory-related candidates by cDNA subtraction, the category of genes predicted to be involved in cellular defense, detoxification, and stress response was the second most prevalent group. For many of these genes, we found differential expression in response to insect herbivory when using hybridization-based profiling methods. Several of these genes were known to respond to insect herbivory from the literature. For example, At3g14210 was only recently identified as *EPITHIOSPECIFIER MODIFIER1* (*ESP1*), a gene controlling Arabidopsis natural genetic variation in glucosinolate hydrolysis product identity and resistance against the cabbage looper (*Trichoplusia ni*) [59]. In addition, we also found a large number of genes not previously described as responding to insect herbivory. For example, sequences from different thionine genes were highly over-represented in our cDNA library obtained with forward suppression subtractive hybridization. Thionins are small cysteine-containing, amphipathic plant proteins found in seeds and vegetative tissues of a number of plant genera. Some of these thionins have been shown to be toxic to pathogens [60]. Thus, they might also have a defensive function in plant-insect interactions. However, functional tests are necessary to elucidate a potential impact on herbivorous lepidopterans.

Our results indicate that transcript patterns in response to insect herbivory and wounding are distinct though partially overlapping. This suggests that other insect cues besides mechanical damage trigger the plant's response to insect herbivory. Such cues could involve chemical compounds associated with the insect surface or with digestive fluids. Indeed, several low- and high-molecular weight compounds have been identified in insect oral secretions, including fatty acid conjugates (FACs) [2], [4], [61] such as volicitin which, when applied to wounded plants, can stimulate *de novo* synthesis and release of volatiles and induction of defense-related genes [3], [62]–[64]. Composition and amount of FACs differs among insects (H. Vogel & D. Spiteller, unpublished results), and some insects, *e.g.*, the tobacco budworm (*Heliothis virescens*), apparently decompose FACs in their midguts, thus controlling the level of defense-inducing compounds and suppressing plant volatile emission [65].

However, most experiments (including the present study) that contrast herbivory from wound-related plant responses have mimicked mechanical damage exerted by insects only imperfectly, preventing a clear separation of wound- and chemical-elicited components in the plant's response to insect herbivory. Therefore, a more precise imitation of insect behavior during herbivory will be necessary, allowing imitation of both the pattern and amount of damage in a quantifiable and repeatable manner. Suitable devices have been developed [66], and will be used in future transcript profiling experiments relating to insect herbivory.

### Differential Responses to Two Herbivorous Lepidopteran Insects

Reymond *et al.* [22] investigated *A. thaliana* transcript patterns in response to two different herbivorous lepidopterans, the specialist *P. rapae* and the generalist *S. littoralis*, and they identified more than 100 insect-responsive genes. Surprisingly, plant transcriptional responses to both insects were remarkably similar, with a large overlap in the sets of genes differentially expressed upon *P. rapae* or *S. littoralis* herbivory. With both insects the vast majority of genes were induced and very few repressed. Furthermore, utilizing mutants impaired in the JA pathway, Reymond *et al.* [22], concluded that the majority of insect-responsive genes were under the control of JA, emphasizing a predominant role of this phytohormone in mediating plant responses to insect herbivory. By contrast, De Vos *et al.* found that there was, in general, fairly little overlap in Arabidopsis transcript patterns in response to insects with different feeding modes [35]. They also found that JA played a prominent role in the interaction between Arabidopsis and leaf-chewing *P. rapae* or cell-content-feeding thrips, *Frankliniella occidentalis*, but not in the interaction with phloem-feeding aphids, *Myzus persicae*. These different results may, of course, be attributable to differences in insect feeding mode or could simply reflect a different degree of evolutionary relatedness between the insect species investigated in both studies.

In our study, we investigated plant transcript patterns in response to two further lepidopteran herbivores, the specialist *P. xylostella*, and the generalist *T. ni*. A large number of genes had higher transcript abundance after *P. xylostella* herbivory compared to wounded or control plants, but we also identified a substantial number of repressed genes. Many genes identified as insect-responsive in our study were not identified in the cDNA microarrays used by Reymond *et al.* [22]. On the other hand, several differentially expressed genes from Reymond *et al.* [22] were not significantly regulated in our experiments. Clearly, many factors differ between Reymond *et al.* [22] and the current study, including array platforms, statistical procedures, and experimental conditions. Nonetheless, these comparisons suggest that transcript profiles after *P. xylostella* herbivory differ from those after feeding by *P. rapae* or *S. littoralis*. Furthermore, our macroarray is enriched with *P. xylostella*-responsive genes which could introduce a certain bias to our analyses. Nonetheless, a large number of genes that are differentially regulated after *P. xylostella* herbivory do not respond to *T. ni*. In fact, our analysis is conservative, because if a *P. xylostella*-based array obscures differences between both insect species, then the differences are even bigger than what we present.

To more directly address the question whether different lepidopteran insects trigger different plant responses, we used path analysis, a method novel to the analysis of expression profiling data. Since we found very similar transcript patterns between *A. thaliana* and *B. divaricarpa* across a variety of different experimental treatments, we focused this analysis on *B. divaricarpa*. For this species, we had performed experiments with both insects, *P. xylostella* and *T. ni*, wounding, and exogenous application of JA, SA, and ethylene, in addition to unmanipulated controls.

Induced responses to *T. ni* showed greatest similarity to ethylene and JA-induced changes. In contrast, transcriptional responses to *P. xylostella* were best predicted by ethylene and SA-related changes. Hence, path analysis reveals that regulatory control and transcriptional responses to insect feeding are very different between these two lepidopteran herbivores (Figure 3). Finally, responses to wounding showed similarity to all three hormonal pathways. These findings suggest that insects interfere actively with the plants' response to herbivory. However, further work is required to determine the factors that cause these differential responses.

## Supporting Information

Table S1 TAIR identifiers corresponding to best BLAST hits from the B. divaricarpa subtractive cDNA library and their GO annotation.
(4.17 MB XLS)

Table S2 Custom macroarray setup. Worksheet 1 gives information on unambiguous setup identifiers (‘Clone Plate ID’), spot numbers (‘SPOT No.’), the original feature name (‘Clone Name’), the GenBank accession number (‘Accession No.’), its source (‘Species’, either B. divaricarpa or A. thaliana), TAIR identifiers (when possible), together with a description of the ‘Best BLAST Hit’ or other information, ‘E-values’ as an indicator for match quality, cDNA ‘Sequence’ and ‘Size’. For controls, no best BLAST matches, E-values, sequences, or sizes are given. Worksheet 2 contains information on the dilution series of RNA spikes corresponding to ALIEN cDNAs used for data normalization after hybridization. Worksheets 3 and 4 contain information on the plate setups for two 384-well plates containing samples for spotting of the array. Each feature is represented by ‘Clone Plate IDs’ (see above). Worksheet 5 contains information on the final spot pattern.
(0.50 MB XLS)

Table S3 Custom macroarray data. Custom array identifiers (‘Clone plate ID’), and the original feature name (‘Clone name’) are given in the first two columns. All other columns contain hybridization data. Abbreviations are as follows: AT, hybridization with cDNA obtained from A. thaliana; BD, hybridization with cDNA obtained from B. divaricarpa; C, control; W, wounding; ID, herbivory by P. xylostella; IT, herbivory by T. ni; E, ethylene treatment; JA, jasmonic acid treatment; SA, salicylic acid treatment. Timepoints used in these experiments are 45 minutes (45), 3, 9, and 24 hours (3, 9, 24, respectively). Number/letter combinations (e.g., 1a, 2a, etc.) after the underscore indicate independent biological replicates of a given treatment. Data normalization and transformation procedures are described in MATERIALS AND METHODS.
(3.55 MB XLS)

Table S4 cDNAs with significant differential regulation in B. divaricarpa experiments. ‘Gene’ refers to pairs of features on the custom macroarray, while the ‘TAIR identifier’ (www.arabidopsis.org) is based on the best BLAST match of a given sequence from this macroarray. ‘Source’ gives the origin of a cDNA, together with a description of the corresponding gene. ‘F-RatioTRT’ quantifies statistical significance for differences in transcript levels between treatments. Furthermore, normalized data for controls (‘C’), wounding (‘W’), herbivory by P. xylostella (‘ID’), herbivory by T. ni (‘IT’), ethylene (‘E’), jasmonic acid (‘JA’), and salicylic acid (‘SA’) treatments are given for t = 45 min (‘45’), 3 hours (‘3’), 9 hours (‘9’), and/or 24 hours (‘24’). Data normalization and transformation procedures are described in MATERIALS AND METHODS. ‘ID9/W9’ and ‘IT9/W9’ indicate transcript level ratios for herbivory by P. xylostella or T. ni vs. wounding at 9 hours.
(0.15 MB XLS)

Table S5 Cross species comparison for differences in transcript levels and patterns between A. thaliana and B. divaricarpa. ‘GenePair’ refers to pairs of array features representing the same B. divaricarpa and A. thaliana cDNAs, based on best BLAST matches. ‘TRT2SPP$’, ‘HOMOHET$’, ‘MRNASPP$’, and ‘MRNA*TRT’ are P-values for effects in ANOVA, and quantify statistical significance for treatment effects, for differences between homologous vs. heterologous transcript-target hybridizations, for mean transcript levels between species, and for species × treatment interaction, respectively. ‘HET’ and ‘HOMO’ are signal intensities from heterologous and homologous transcript-target hybridizations, ‘HOMO/HET’ is their ratio. ‘Qval_MRNASPP$’ and ‘Qval_MRNA*TRT’ indicate Q-values for gene × species and gene × treatment effects, respectively. ‘Qval_MRNASPP$’ quantifies statistical significance for differences in mean transcript levels between species, ‘Qval_MRN*TRT’ quantifies statistical significance for differences in transcript patterns between species. Bold type letters indicate statistically significant differences in mean transcript level or in transcript patterns between species according to P- and Q-statistics (P≤0.05 and Q≤0.05). ‘LSM (At)’ and ‘LSM (Bd)’ are least square means for signal intensities, averaged across treatments. SE indicates standard errors, and N sample sizes. ‘At/Bd’ and Bd/At’ quantify ratios of mean signal intensities between species, and ‘((Bd/At)−1)*100%’ quantifies relative differences in mean transcript level between species.
(0.04 MB XLS)
